# Categorize the existing clamps used for tensile test of human graft– a systematic review

**DOI:** 10.1186/s12891-022-05650-w

**Published:** 2022-07-25

**Authors:** Denes Farago, Blanka Kozma, Rita Maria Kiss

**Affiliations:** 1 Cooperation Research Center for Biomechanics, Faculty of Mechanical Engineering, Budapest University of Technology and Economics, Budapest, Hungary; 2 Department of Mechatronics, Optics and Mechanical Engineering Informatics, Faculty of Mechanical Engineering, Budapest University of Technology and Economics, Budapest, Hungary; 3 Department of Biomedical Engineering, SUNY University at Buffalo, Buffalo, USA

**Keywords:** Tendon, Biomechanical endurance test of tendon, Clamp type, Mechanical properties

## Abstract

**Background:**

The use of tendon allografts for orthopedic repair has gained wide acceptance in recent years, most notably in anterior cruciate tendon reconstruction. Multiple studies support the use of tendon allografts and the benefits of its use are well accepted and understood. One of the important criteria of the use of tendon allografts is statistically similar histological and biomechanical properties to autographs. The aim of this systematic literature review is to investigate and categorize existing clamps used in the determination of the biomechanical properties of tendons such as maximum load, maximum strength, modulus of elasticity, ultimate strain, and stiffness. A variety of clamps for use during the endurance test of tendons were categorized according to the temperature used during the measurement. The clamps are divided into three groups: room temperature, cooled and heated clamps. The second goal of our review is to overview of clamps on the following aspects: name of clamp, author and date, type of clamps, type of endurance test (static or dynamic), type preloading (dynamic or static), type of tendon and measured and calculated parameters, and summarize in Table 3, as a comprehensive catalogue.

**Methods:**

This systematic review was carried out in keeping with the PRISMA 2020 E&E and the PRISMA-S guidelines and checklists. A search was conducted for publications dating between 1991 and February 28th 2022 through three electronic databases (Web of Science, Scopus, and PubMed). We used Critical Appraisal Skills Program checklist to check the quality of included articles.

**Results:**

The database search and additional sources resulted in 1725 records. 1635 records eliminated during the screening for various reasons (case report, other languages, book chapter, unavailable text/conference abstract, unrelated topic). The number of articles used in the final synthesis was 90. A variety of clamps for use during the endurance test of tendons were identified and categorized according to the temperature used during the measurement. Based on this, the clamps are divided into three groups: room temperature, cooled or heated clamps.

**Conclusions:**

On the basis of the systematic literature review, mechanical parameters determined by usage with cooled clamps proved to be more reliable than with those at room temperature and with heated clamps. The collected information from the articles included name of clamp, author and date, type of clamps, type of endurance test (static or dynamic), type preloading (dynamic or static), type of tendon and measured and calculated parameters given in Table 3. summarized. The main advantage of the cooled clamps is that there is no limit to the type and length of the tendon. This study provides an overview of clamps and does not represent the modernity of any method.

**Supplementary Information:**

The online version contains supplementary material available at 10.1186/s12891-022-05650-w.

## Introduction

The use of tendon allografts for orthopedic repair has gained wide acceptance in recent years, most notably in anterior cruciate tendon reconstruction [1–3]. Multiple studies support the use of tendon allografts and the benefits of its use are well accepted and understood [2, 4–7]. Specifically, these benefits include decreased surgical time, decreased surgical morbidity and unaltered mechanics secondary to harvesting. Furthermore, animal and human studies have shown that soft tissue allografts are statistically similar to autografts on a histological and biomechanical basis [8–10].

Anterior cruciate ligament (ACL) reconstruction is a common procedure in orthopedic practice. One of the most important decisions for the surgeon to make is the right choice of graft. Although autografts have proven to be capable and showed good clinical outcomes, graft harvest can cause persistent pain at the harvest site and a limited range of motion [11–14]. Therefore, allograft use has significantly increased in the last decades. Since it eliminates donor-site morbidity, and albeit its use is associated with higher costs, it remains a viable option, especially in revision cases. In order to ensure that there is a minimal biomechanical difference between the ACL and the graft, the biomechanical properties need to be tested so that we can choose which tendons can be good substitutes [7, 15]. The tendons are subjected to tensile testing, which can be static or dynamic. From these we get a force-elongation diagram, which can be calculated based on, for example the Young’s modulus of elasticity [16–18].

The purpose of a clamp is a proper fixation technique for allograft endurance tests, and adapt it to be compatible for the loading machine [10, 19]. The main problem with tendon clamps is that it is hard to maintain the high pressure needed to provide enough friction force between the tendon and the clamp to resist a large tensile load, and at the same time to reduce the cutting effect of the clamp, reducing slippage danger [7, 20–24].

Various clamps have been developed for the assessment of the endurance test. These clamps are usually specific for measurement methods, thus, the results of the measurement methods are difficult to compare [1, 8, 11–15, 25, 26].

### Aim of study

The literature of the effect of the sterilization method on the material properties of the tendon is well researched and discussed [27–31]. Nevertheless, there are no systematic reviews on the subject that would provide guidance on the clamps used for the measurements. The aim of this systematic literature review is to investigate and categorize existing clamps used in the determination of the biomechanical properties of tendons such as maximum load, maximum strength, modulus of elasticity, ultimate strain, and stiffness. A variety of clamps for use during the endurance test of tendons were categorized according to the temperature used during the measurement. The clamps are divided into three groups: room temperature, cooled and heated clamps. The second goal of our review is to overview of clamps on the following aspects: name of clamp, author and date, type of clamps, type of endurance test (static or dynamic), type preloading (dynamic or static), type of tendon and measured and calculated parameters, and summarize in Table 1, as a comprehensive catalogue.

**Table 1:** Inclusion and exclusion criteria

Viewpoints	Inclusion	Exclusion
**Tendon and endurance test and clamp**	Studies which included tendon and endurance test and clamp in their experimental procedures.	Studies which only included a tendon measurement method without any type of clamp.
**Description of tendon and endurance test and clamp**	Studies with detailed descriptions of the tendon and endurance test and clamp and the experimental process that was followed.	Studies without detail or incomplete descriptions of the clamp and endurance test and the experimental process that was followed.
**Assessment of results**	Studies with objective result assessment based on measurable parameters.	Studies with subjective scoring/assessment of results, not (entirely) based on measurable parameters.

## Materials and methods

### Data sources and search strategy

This systematic review was carried out in keeping with the PRISMA 2020 E&E and the PRISMA-S guidelines and checklists [32, 33]. A search was conducted for publications dating between 1991 and February 28th 2022 through three electronic databases (Web of Science, Scopus, and PubMed). The searches were conducted on March 1st 2022.

The electronic search for the Web of Science database is shown below. These terms were added into the Advanced search option, using the ‘All fields’ option: ALL=((allograft tendon OR allograft tendon* OR (allograft* AND tendon*)) AND (biomechanical pull-out test* OR stiffness OR strength OR mechanical properties OR modulus OR endurance test* OR clamp OR clamps OR clamp*)). The search was limited to journal publications. Publication date limits were set to from 1991, with the search performed on February 28th, 2022. The search of the Web of Science database yielded 670 records.

The Scopus database was searched as follows. Were used the basic search, in ‘Search within’ were used ‘All fields’ option. In ‘Search documents’ were used the follow search strategy: (allograft OR tendon) AND (biomechanical AND pull-out AND test OR stiffness OR strength OR mechanical AND properties OR modulus OR endurance AND test* OR clamp OR clamps). The search of the Scopus database yielded 599 records.

The PubMed database was searched as follows. These terms were added into the ‘Advanced’ option, using ‘All fields’ and were used to the ‘Query box’ the follows: ((“allograft tendon“[tw] OR “allograft tendons“[tw] OR (allograft* AND tendon*)) AND (“biomechanical pull-out test*“[tw] OR “stiffness“[tw] OR “strength*“[tw] OR “mechanical propert*“[tw] OR “modulus“[tw] OR “endurance test*“[tw] OR clamp[tw] OR clamps[tw] OR clamp*[tw])) AND (“1992/01/01“[PDAT] : “2022/02/28“[PDAT]). The search of the PubMed database yielded 456 records.

Key search terms were identified and agreed upon by DF and RMK; electronic search and downloading of results were conducted by DF. Screening, eligibility check of materials and date extraction were carried out by DF and BK [34]. The reviewers worked independently and no automation tools were used at each stage of screening. Our search strategy excludes examines based on a reference list.Screening materials.

### Screening materials

After removing the duplicates, the identified publications were screened based on their title and their abstracts. Publications of exclusively theoretical work or included studies of purely theoretical work or with topics deviating from the aim of study were excluded.

### Inclusion and exclusion criteria

In order to confirm eligibility for the study, the reviewers defined the inclusion and exclusion criteria. The publications had to meet each inclusion criterion to be incorporated in the final synthesis (Table 2). If a study failed to meet any inclusion criteria, or met an exclusion criterion, it was excluded. The criteria were carefully chosen to ensure a quality assessment of the material to a certain extent, i.e., the methods used had to be well communicated and the evaluation of measurement results had to be objective.

**Table 2:** Results of quality assessment for each included article. Yes: 1; No: 0; Can’t Tell: 2

	Was there a clear statement of the aims of the research?	Is a qualitative methodology appropriate?	Was the research design appropriate to address the aims of the research?	Was the recruitment strategy appropiate to the aims of the research?	Was the data collected in a way that addressed the research issue?	Has the relationship between researcher and patricipants been adequately considered?	Have ethical issues been taken into consideration?	Was the data analysis sufficiently rigorous?	Is there a clear statement of findings?	How valuable is the research?	Overall quality assessment
Aeberhard 2019 [35]	1	1	1	1	1	1	1	1	1	1	high
Aguila 2016 [36]	1	1	1	2	1	1	1	1	1	1	high
Athwal 2020 [37]	1	1	1	1	1	1	1	1	1	1	high
Awogni 2014 [38]	1	1	1	1	1	1	1	1	1	1	high
Aynardi 2017 [39]	1	1	1	1	1	1	1	1	1	1	high
Azar 2009 [40]	1	1	1	1	1	1		1	1	1	high
Bachmaier 2020 [41]	1	1	1	1	1	1	1	1	1	1	high
Baer 2007 [6]	1	1	1	1	1	1	1	1	1	1	high
Baldini 2014 [42]	1	1	1	1	1	1	1	1	1	1	high
Balsly 2008 [43]	1	1	1	1	1	1	1	1	1	1	high
Barros 2021 [44]	1	1	1	1	1	1	1	1	1	1	high
Bartolo 2021 [45]	1	1	1	1	1	1	1	1	1	1	high
Basso 2002 [46]	1	1	1	1	1	1	1	1	1	1	high
Bechtold 1994 [47]	1	1	1	1	1	1	1	1	1	1	high
Berlet 2014 [48]	1	1	1	1	1	1	1	1	1	1	high
Bernstein 2022 [49]	1	1	1	1	1	1	1	1	1	1	high
Bi 2018 [50]	1	1	1	1	1	1	1	1	1	1	high
Braunstein 2015 [51]	1	1	1	1	1	1	1	1	1	1	high
Chivot 2017 [52]	1	1	1	1	1	1	1	1	1	1	high
Chizari 2011 [53]	1	1	1	1	1	1	1	1	1	1	high
Colaco 2017 [54]	1	1	1	1	1	1	1	1	1	1	high
Coleridge 2004 [55]	1	1	1	1	1	1	1	1	1	1	high
Conrad 2012 [10]	1	1	1	1	1	1	1	1	1	1	high
Curran 2004 [56]	1	1	1	1	1	1	1	1	1	1	high
Delgado 2014 [27]	1	1	1	1	1	1	1	1	1	1	high
Dibartola 2016 [30]	1	1	1	1	1	1	1	1	1	1	high
Dyrna 2018 [57]	1	1	1	1	1	1	1	1	1	1	high
Dziedzic-Goclawska 2005 [58]	1	1	1	1	1	1	1	1	1	1	high
Edwards 2016 [59]	1	1	1	1	1	1	1	1	1	1	high
Ehrensberger 2013 [60]	1	1	1	1	1	1	1	1	1	1	high
Elenes 2014 [61]	1	1	1	1	1	1	1	1	1	1	high
Erivan 2018 [62]	1	1	1	1	1	1	1	1	1	1	high
Farago 2020 [63]	1	1	1	1	1	1	1	1	1	1	high
Gaines 2017 [64]	1	1	1	1	1	1	1	1	1	1	high
Gardner 2013 [65]	1	1	1	1	1	1	1	1	1	1	high
Giannini 2008 [66]	1	1	1	1	1	1	1	1	1	1	high
Gibbons 1991 [67]	1	1	1	1	1	1	1	1	1	1	high
Goh 2014 [19]	1	1	1	1	1	1	1	1	1	1	high
Gokler 2021 [68]	1	1	1	1	1	1	1	1	1	1	high
Greaves 2008 [69]	1	1	1	1	1	1	1	1	1	1	high
Guerroudj 2007 [70]	1	1	1	1	1	1	1	1	1	1	high
Gut 2015 [71]	1	1	1	1	1	1	1	1	1	1	high
Halewood 2011 [72]	1	1	1	1	1	1	1	1	1	1	high
Hangody 2016 [73]	1	1	1	1	1	1	1	1	1	1	high
Hangody 2017 [74]	1	1	1	1	1	1	1	1	1	1	high
Hashemi 2005 [75]	1	1	1	1	1	1	1	1	1	1	high
Herbert 2017 [76]	1	1	1	1	1	1	1	1	1	1	high
Hoburg 2010 [77]	1	1	1	1	1	1	1	1	1	1	high
Hoburg 2011 [78]	1	1	1	1	1	1	1	1	1	1	high
Hoburg 2014 [79]	1	1	1	1	1	1	1	1	1	1	high
Höher 2013 [80]	1	1	1	1	1	1	1	1	1	1	high
Huang 2013 [81]	1	1	1	1	1	1	1	1	1	1	high
Irani 2018 [82]	1	1	1	1	1	1	1	1	1	1	high
Jones 2007 [83]	1	1	1	1	1	1	1	1	1	1	high
Jung 2011 [84]	1	1	1	1	1	1	1	1	1	1	high
Kemper 2010 [85]	1	1	1	1	1	1	1	1	1	1	high
Kranjec 2020 [86]	1	1	1	1	1	1	1	1	1	1	high
Lansdown 2017 [28]	1	1	1	1	1	1	1	1	1	1	high
Lenschow 2014 [87]	1	1	1	1	1	1	1	1	1	1	high
Mae 2003 [8]	1	1	1	1	1	1	1	1	1	1	high
Mahirogullari 2007 [9]	1	1	1	1	1	1	1	1	1	1	high
McGilvary 2010 [88]	1	1	1	1	1	1	1	1	1	1	high
Miller 2017 [89]	1	1	1	1	1	1	1	1	1	1	high
Mook 2017 [90]	1	1	1	1	1	1	1	1	1	1	high
Ng 2012 [91]	1	1	1	1	1	1	1	1	1	1	high
Ninomiya 2011 [92]	1	1	1	1	1	1	1	1	1	1	high
Oswald 2017 [93]	1	1	1	1	1	1	1	1	1	1	high
Pailhé 2015 [94]	1	1	1	1	1	1	1	1	1	1	high
Penn 2009 [95]	1	1	1	1	1	1	1	1	1	1	high
Proberaj 2020 [96]	1	1	1	1	1	1	1	1	1	1	high
Rasmussen 1994 [97]	1	1	1	1	1	1	1	1	1	1	high
Ren 2012 [98]	1	1	1	1	1	1	1	1	1	1	high
Roberson 2017 [31]	1	1	1	1	1	1	1	1	1	1	high
Rudy 2017 [99]	1	1	1	1	1	1	1	1	1	1	high
Salehpour 1995 [100]	1	1	1	1	1	1	1	1	1	1	high
Samsell 2011 [14]	1	1	1	1	1	1	1	1	1	1	high
Schimizzi 2007 [101]	1	1	1	1	1	1	1	1	1	1	high
Schmidt 2012 [102]	1	1	1	1	1	1	1	1	1	1	high
Schmidt 2016 [13]	1	1	1	1	1	1	1	1	1	1	high
Schmidt 2019 [103]	1	1	1	1	1	1	1	1	1	1	high
Seto 2012 [104]	1	1	1	1	1	1	1	1	1	1	high
Smith 1996 [105]	1	1	1	1	1	1	1	1	1	1	high
Sobel 2012 [106]	1	1	1	1	1	1	1	1	1	1	high
Suhodolcan 2012 [107]	1	1	1	1	1	1	1	1	1	1	high
Swank 2014 [108]	1	1	1	1	1	1	1	1	1	1	high
Tse 2012 [109]	1	1	1	1	1	1	1	1	1	1	high
Weber 2018 [110]	1	1	1	1	1	1	1	1	1	1	high
Yanke 2013 [100]	1	1	1	1	1	1	1	1	1	1	high
Yanke 2013-2 [111]	1	1	1	1	1	1	1	1	1	1	high

### Data extraction and analysis

In accordance with the focus of this review, the final synthesis of the collected types of clamps included extracted relevant information on the evaluation of mechanical properties. The collected information from the articles included: name of clamp, author and date, type of clamps, type of endurance test (static or dynamic), type preloading (dynamic or static), type of tendon and measured and calculated parameters.

### Study quality, risk of Bias

Articles were evaluated using the Critical Appraisal Skills Program (CASP) quality assessment tool [112]. CASP contains several checklists, one of which is the CASP Qualitative Studies Checklist of 10 questions that we used. This checklist has several items that allow authors to rate articles for “low”, “medium” and “high” quality assessment. This review is by two authors (DF and RMK) and active discussion until consensus was reached in the case of rating discrepancies. We did not undertake a risk of bias assessment because the included studies were not randomized controlled studies and because our evidence synthesis method is outside of systematic reviews.

## Results

The search of the database source gave 1725 results (Prisma 2020 Flow Diagram). Removing duplications 1361 literatures remained. When screening the titles and the abstracts, an additional 657 records were excluded, due to not fitting the scope. The remaining 704 articles have been read in their entirety. Of these studies, 567 were excluded with justifications of not meeting the eligibility criteria (without any type of clamp, incomplete description, subjective results). These review articles had a different scope from our current study. The number of articles included in the final synthesis was 90 (*n *= 90). The flow diagram describing the process has uploaded as a Supplementary file1.

Table 3 summarizes the results of the quality assessment for each included article. One articles [113] had an inadequate recruitment strategy. All other articles were rated “high” in all respects.

**Table 3:** Overview of clamps as a comprehensive catalogue

Name of clamp	References	Type of clamp	Type of endurance test	Pre- loading type	Type of tendon	Measured and calculated parameters
Metal U-shaped frames	47, 50	room temperature	static	dynamic	sheep patellar tendon	failure stress, failure strain, normalized stiffness, energy to failure
Custom designed clamps	67	room temperature	static	static	canine patella-ligament-tibia	failure load, stiffness
Factory clamps	36	room temperature	dynamic	dynamic	human patellar tendon	ultimate elongation, ultimate stress, ultimate stiffness
Wedge shaped factory-clamps	42	room temperature	dynamic	static	achilles	maximum stress, maximum strain, modulus
Wedge-grip clamps	34, 38	room temperature	dynamic	dynamic	human patellar tendon	failure load, stiffness
Aluminum grips with polymer liners	40, 59, 60	room temperature	dynamic	dynamic	human patellar tendon	failure load, stiffness, strain
Testing configuration for single-strand and double-strand	32, 69	cooled temperature	static and dynamic	dynamic	tibialis anterior and posterior	linear stiffness, ultimate tensile force, tensile modulus, ultimate tensile strength, ultimate tensile strain
Custom designed clamps with dry ice chamber	28	cooled temperature	dynamic	dynamic	anterior and posterior tibialis	failure load, failure stress, stiffness
Factory clamps with dry ice chamber	56	cooled temperature	dynamic	dynamic	achilles, quadriceps, semitendinosus + gracilis, tibialis anterior, peroneus longus	Young’s modulus of elasticity, maximum load, strain at tensile strength, strain at break
Clamp with thermocouple	37	heated temperature	dynamic	dynamic	bilateral patellar tendon	tensile strength, tensile modulus
Custom clamp in testing chamber	57	heated temperature	static and dynamic	static and dynamic	human patellar tendon	stiffness, maximum load
Custom clamp in biochamber	70	heated temperature	dynamic	dynamic	soleus tendon	ultimate tensile stress, elastic modulus, toughness

### Type of clamps

The systematic review aimed at creating a comprehensive catalogue of existing clamps used in the determination of biomechanical properties. These studies evaluated what kind of impact the type of clamp had on the measurement [35–39, 41–46, 48–53, 55–57, 59–66, 68–82, 84–87, 89, 90, 92–96, 98, 99, 101–104, 106–111, 113–117].A variety of clamps for use during the endurance test of tendons were categorized according to the temperature used during the measurement. The clamps are divided into three groups: room temperature clamps [61, 106, 107] [35, 37–39, 41, 44–46, 48, 49, 51–53, 55–57, 59, 62, 64, 70, 72, 75, 77–80, 84, 85, 87, 89, 90, 92–94, 96, 98, 99, 101–103, 109–111, 115–117], cooled clamps (under room temperature with ice, cooled air, dry ice or liquid nitrogen) [36, 42, 43, 60, 63, 65, 66, 68, 69, 73, 74, 76, 82, 95, 108] and heated clamps (over room temperature with heated air, heated fluids) [50, 81, 86, 104, 113, 114]. All three groups are factory-made and custom-designed clamps.

### Room temperature clamps

Measuring at room temperature is a quick test because it requires the least amount of preparation as there is no need for dry ice, liquid nitrogen, heating, etc. Sufficient force is applied during the measurement to prevent tendon slippage, but no transverse tension is created during the capture of the tissues, which yields invalid results.

**Fig. 1:**
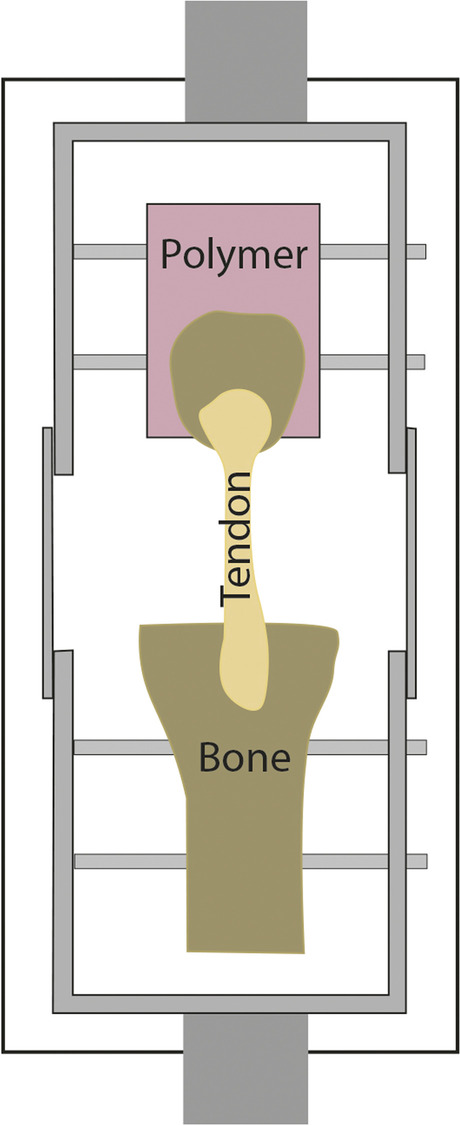
Metal U-shaped frames [115, 116]

**Fig. 2:**
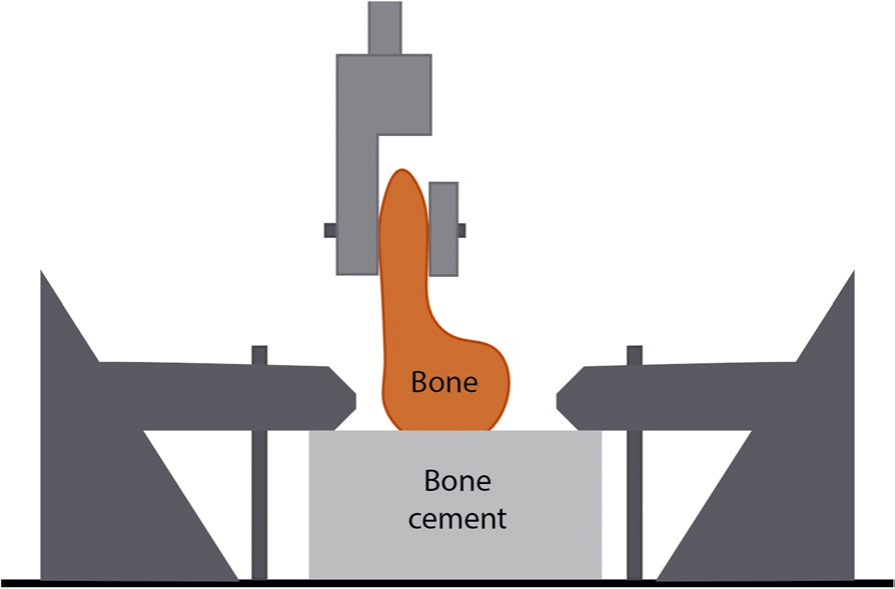
Custom-designed clamps for Canine PLT segments [117]

One of the room temperature clamps is the U-shaped frame (Fig. 1), which can be used for the measurement of the tendon together with the bones. The bone was secured in custom-designed fixation frame with screws. The precision of the drill was ensured by an outer polyethylene mold. [115, 116] In a special case, the bone is inserted into a separately moulded block while the free tendon is pulled by the clamp. The solution allows to investigate the relationship between bone and tendons. (Fig. 2). [117]

**Fig. 3:**
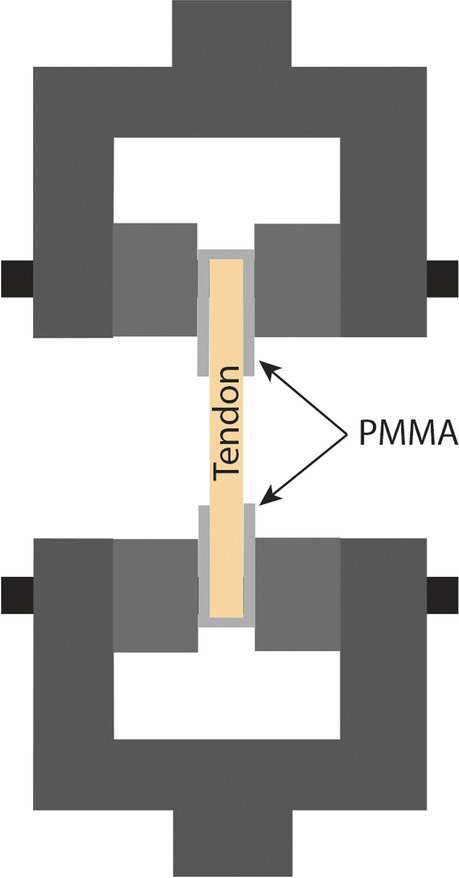
Images of factory clamps (Zwick/Roell) **a**) Osseus blocks potted in polyurethane  fixed into the clamps of the testing device [107]

**Fig. 4:**
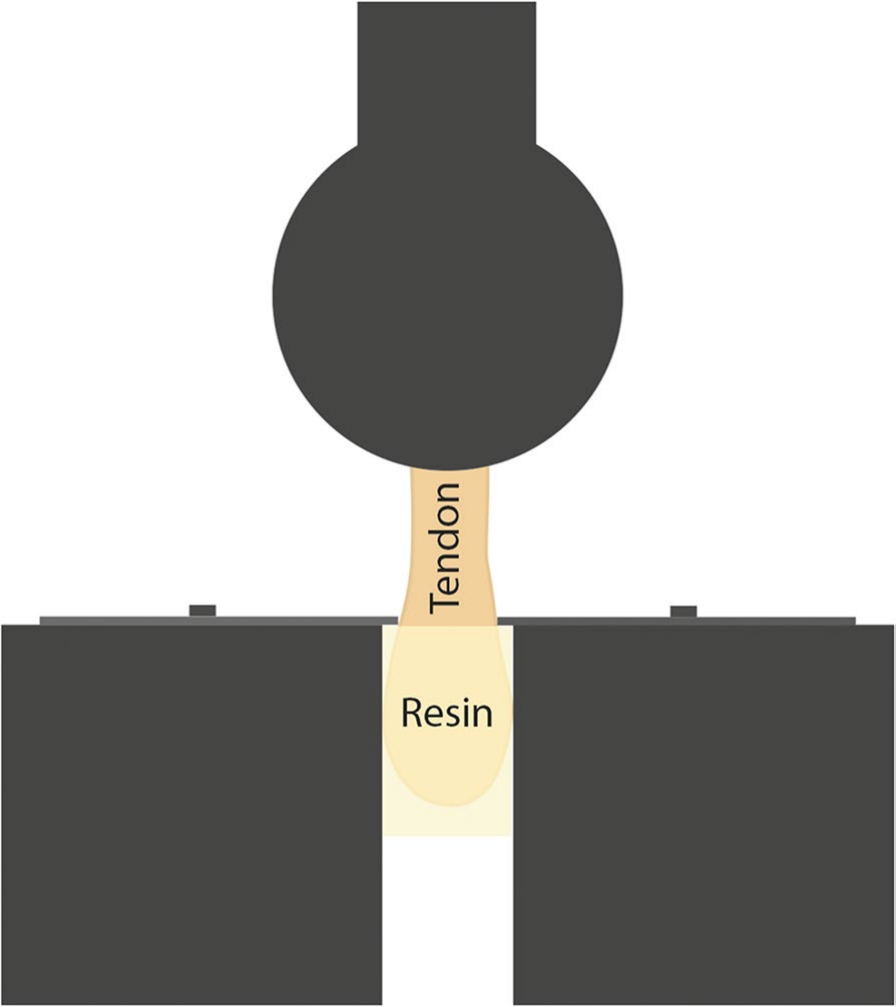
Wedge-shaped factory clamps [110] A special case is when wedge-grip clamp use involves silicone or some kind of artificial resin at both ends to ensure the connection between clamp and tendon [56, 85, 106] (Fig. 5)

Some researchers used custom-designed clamps, where the bone block was secured with either interface polymethylmethacrylate-PMMA or polyurethane [107] (Fig. 3). A solution can also be applied where the natural tendon is fixedby a bone block at one end and by a pneumatic clamp to prevent slippage [110] (Fig. 4). Here, it is particularly important to prevent slippage between the clamp and the tendon, therefore the surface is scratched by sand spraying in several cases.

**Fig. 5:**
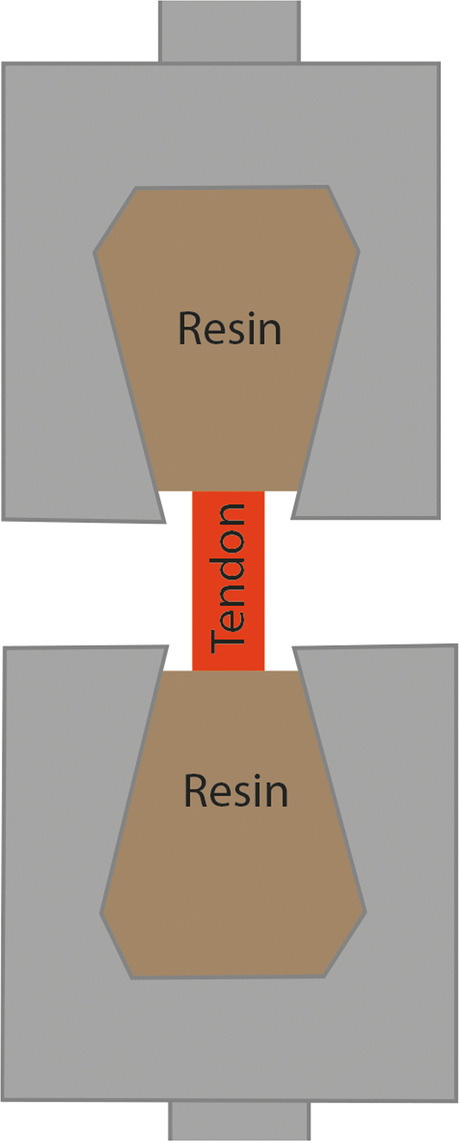
Wedge-grip clamps [56, 106] Several articles use polymer-encapsulated aluminum clamps to achieve better adhesion between the tendon and the clamp (Fig. 6). One of the advantages of the system is that it can be expanded by strain gauges [77–79, 102]

**Fig. 6:**
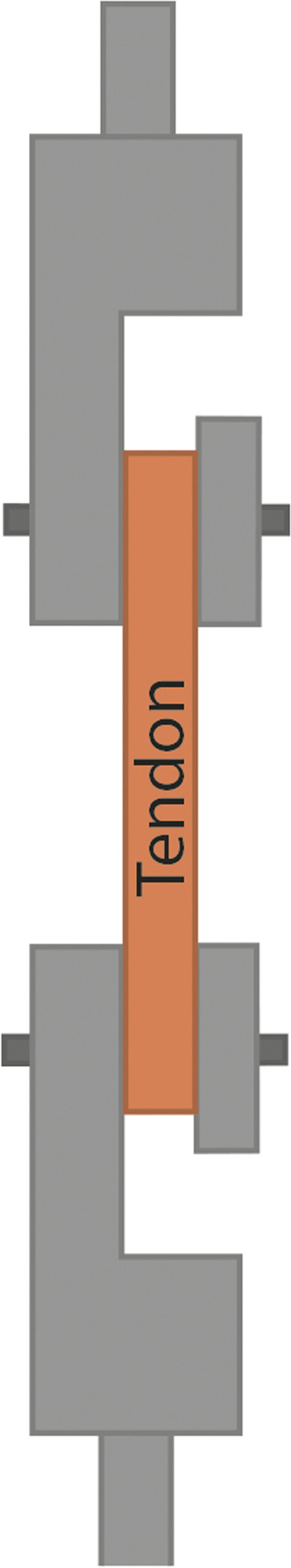
Aluminum grips with polymer liners and strain gauge [77–79] There are articles that do not put any additional material between the ligament and the clamp, using only the factory “serrated” surface of the clamp to prevent slipping (Fig. 7). [35, 62] [49, 93]. [99, 103, 111]

**Fig. 7:**
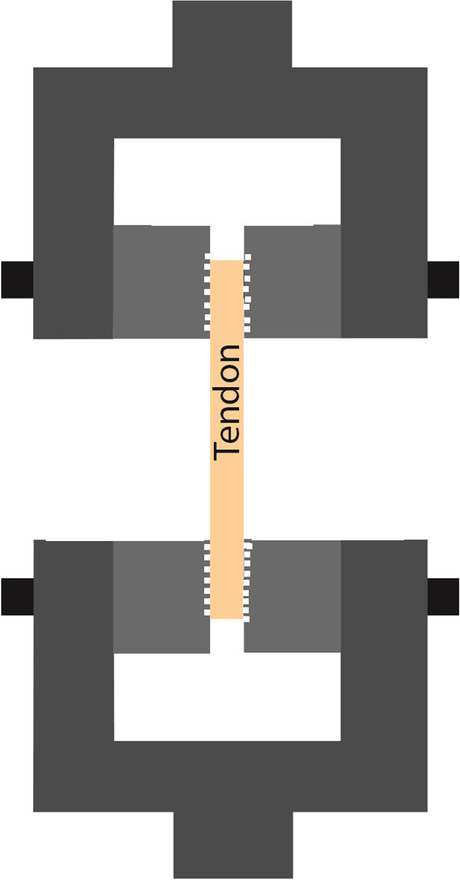
“Serrated” surface [35, 49, 62, 93]. [99, 103, 111]

**Fig. 8:**
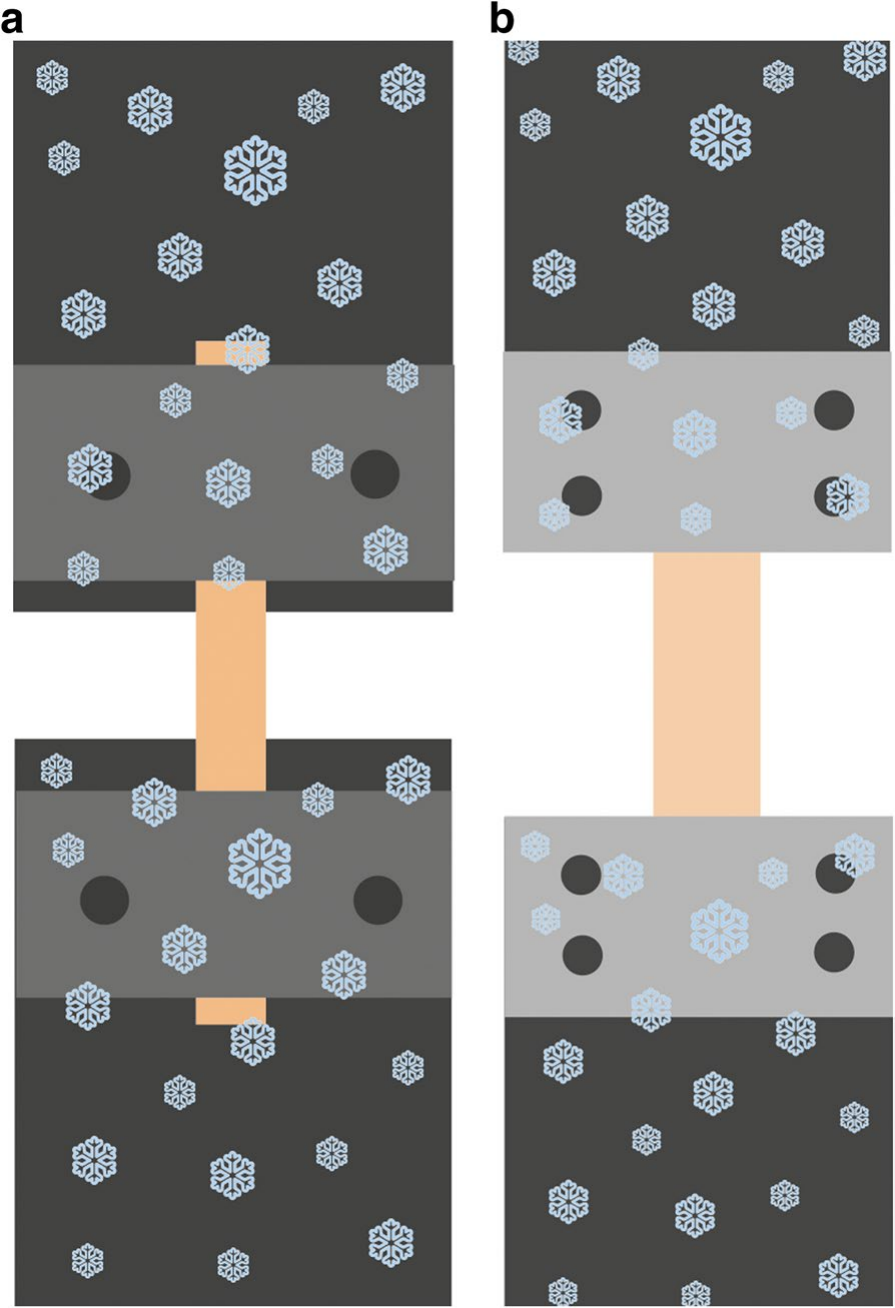
Testing configuration for single-row (**a**) and double-row (**b**) screw fixtures [69, 108]

**Fig. 9:**
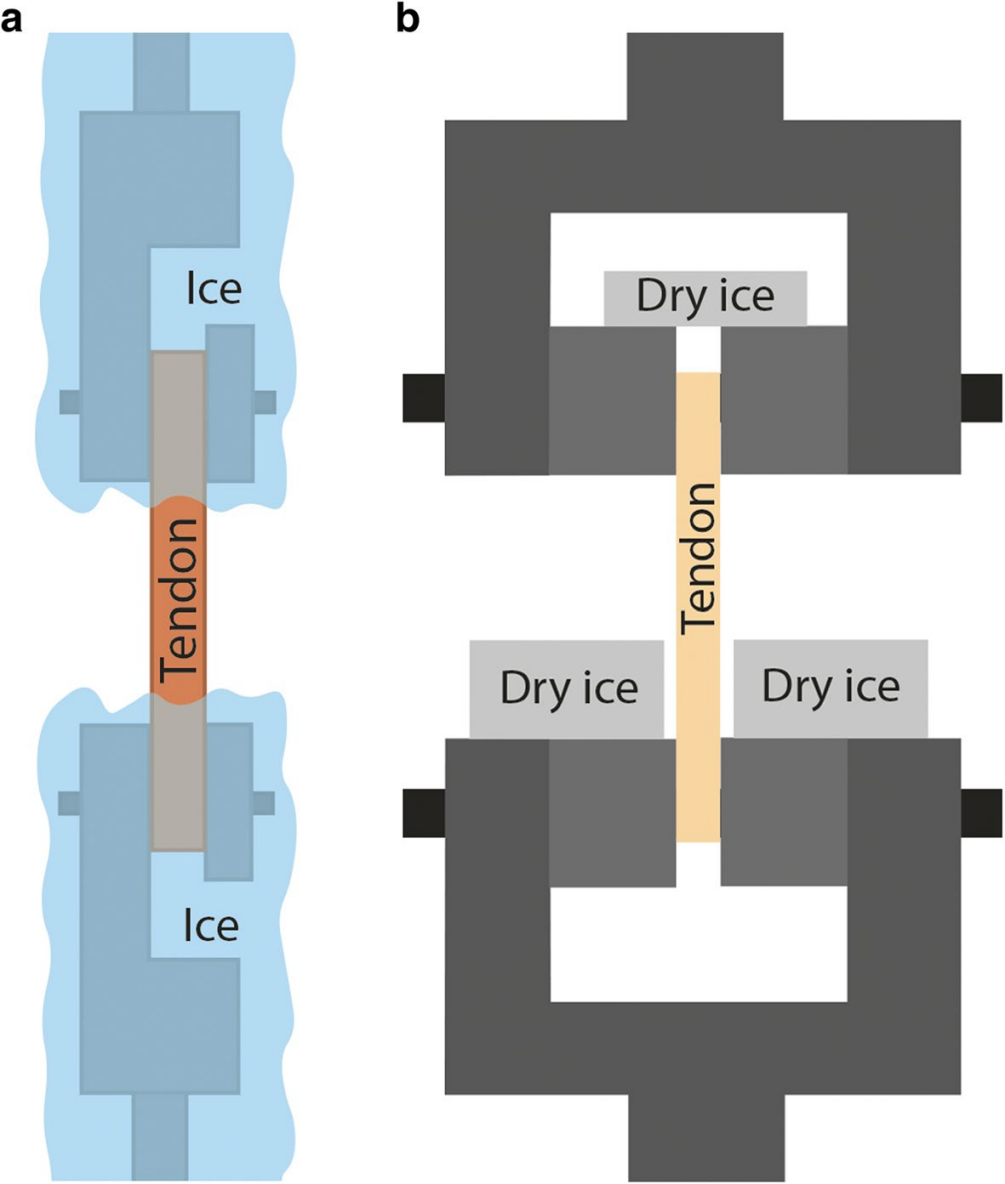
Cooled clamps with different ice chambers **a**) custom-designed clamp [42] **b**) factory clamp [65]

**Fig. 10:**
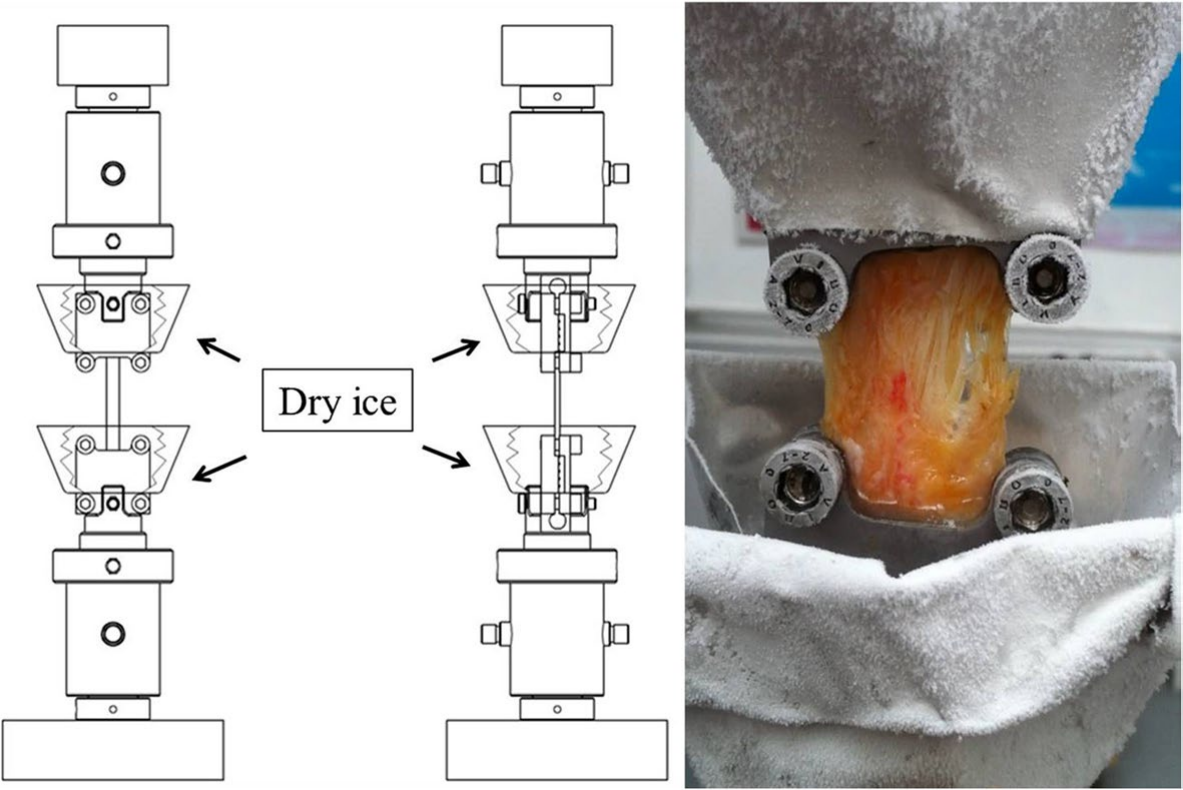
Screwed custom clamps with aluminium chamber for dry ice [73]

**Fig. 11:**
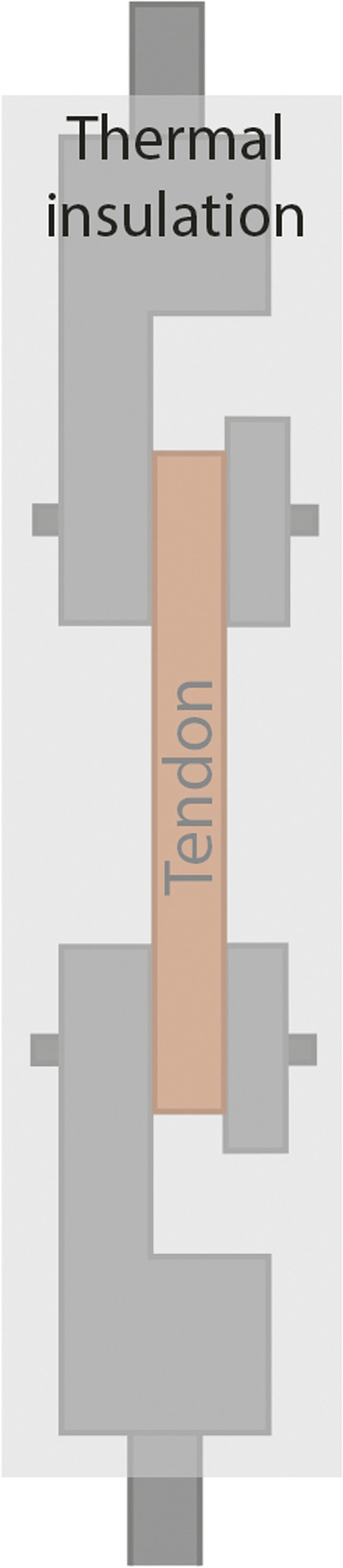
Test device with clamps, insulation, carbon composite rod, load cell, sample and thermocouple [114]

**Fig. 12:**
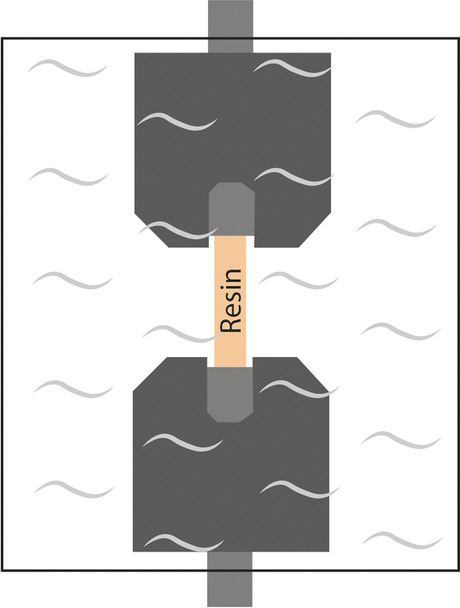
Testing chamber with a PTB specimen mounted in custom grips, showing.eaters used to maintain the phosphate buffered saline at 37°C [81]

**Fig. 13:**
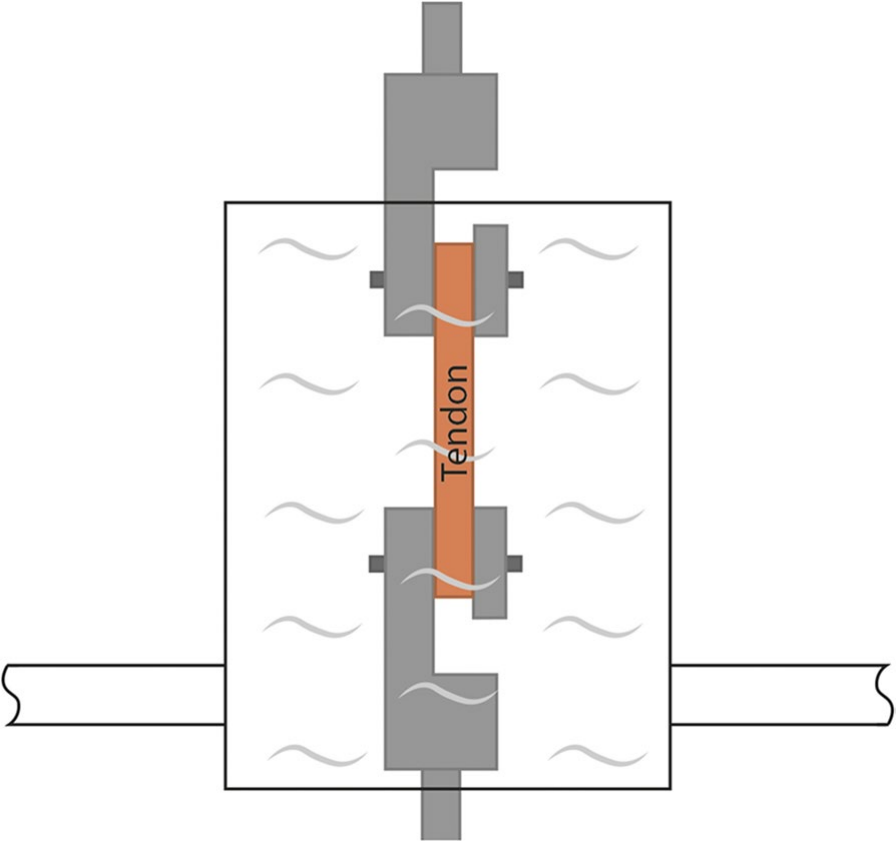
Biochamber used for cyclic loading in solution at 37°C [104]

### Cooled clamps

A basic condition for an appropriate measurement method is to prevent the tendon from slipping out of the clamp, therefore various methods are applied for establishing an adequate connection. One of the reasons for slippage is that the tendon is damp. Therefore it is expedient to continuously freeze the surroundings of the clamp, which naturally scratches the surface. It is expedient to use dry ice or liquid nitrogen for freezing. A disadvantage is that it is not easy to place the freezing substance in the surroundings of the clamp [35–39, 41–46, 48, 49, 51–53, 55, 57, 62, 64, 65, 69, 70, 72, 73, 75, 76, 80, 82, 84, 87, 89, 90, 92–94, 96, 99, 103, 108, 109, 111]. Particular care should be taken that the entire tendon is not completely cooled / frozen because thus the mechanical properties of the tendon are changed. A basic solution for all clamps is that the natural tendon (without the bone) is squeezed between two metal grips, and the two metal grips are fastened to each other by screws. Connection between the grips and the tendon is further increased by grooved metal or plastic inserts fixed on the internal surface of the grips [35–39, 41–46, 48, 49, 51–53, 55, 57, 62, 64, 65, 69, 70, 72, 73, 75, 76, 80, 82, 84, 87, 89, 90, 92–94, 96, 99, 103, 108, 109, 111]. In certain cases, the tendon and the clamp are congealed together, so they work together properly; furthermore, no slippage occurs between tendon and clamp and the tendon does not get torn near the clamp, either [42, 65]. This method can be used in case of tendons of different sizes and types.

However, one of the simplest solutions is that the clams or clamp inserts can be cooled separately before measuring, regardless of the tensile machine. In this case, they should be placed in a deep-freezer for at least 24 h. The tendon is placed into the cooled clamp; the grips squeezing the tendon can be fixed in one or two rows (Fig. 8) [69, 108].

One of the major advantages of cooled clamp use is that factory clamps can be used; it is required to ensure continuous and adequate cooling by placing a chamber of appropriate size to the proper place [42, 65], (Fig. 9). The custom-designed screwed clamp can be made of aluminum plate with a dry ice chamber, where the dry ice can be replaced continuously for ensuring continuous cooling. (Fig. 10) [73].

### Heated clamps

Measurements conducted in an environment of room temperature, using room-temperature or sooled clamps, greatly differ from the temperature of the natural surroundings of tendons (37 °C). Environment temperature presumably affects mechanical properties: more accurate results are yielded if tests are conducted at body temperature. In order to ensure this, it is expedient to use heated clamps [50, 81, 86, 104, 113, 114]. A disadvantage is that, contrary to cooled clamps, the connection between the clamps and the tendon is not improved, but it is also important that it is not deteriorated, either. In general, it is expedient to use a heated liquid for warming [50, 81, 86, 104]; heat insulation should be provided around both the clamps and the component to be examined (Fig. 11) [114]. The measurement can also be performed in a bath filled with heated liquid, which is continuously monitored. It is a basic requirement that the heated liquid should not deteriorate the properties of the tendon (Fig. 12) [81]. The circulation of the liquid simulates the behavior of the blood. (Fig. 13) [104].

## Discussion

The clamp should be designed to prevent the slippage of the tendon from the clamp, but the clamping force should not change the tensile state of the tendon to be examined. The aim of this systematic literature review is to investigate and categorize existing clamps used in the determination of the biomechanical properties of tendons such as maximum load, maximum strength, modulus of elasticity, ultimate strain, and stiffness. A variety of clamps for use during the endurance test of tendons were categorized according to the temperature used during the measurement. The clamps are divided into three groups: room temperature, cooled and heated clamps. The second goal of our review is to overview of clamps on the following aspects: name of clamp, author and date, type of clamps, type of endurance test (static or dynamic), type preloading (dynamic or static), type of tendon and measured and calculated parameters and summarize in Table 1, as a comprehensive catalogue.The clamps are divided into three groups: room temperature, cooled and heated clamps. The collected information from the articles included name of clamp, author and date, type of clamps, type of endurance test (static or dynamic), type preloading (dynamic or static), type of tendon and measured and calculated parameters.The data are summarized in Table 1.

The metal U-shaped frame (Fig. 1) allows for bone-tendon strength to be studied [115, 116]. This clamp also ensures stability of the tendon, not letting it slip out. Because the tendon is clamped tightly, tissue texture can be damaged. In several cases, capture is performed using natural bones (Figs. 1 and 2) or artificial blocks (bone cement, silicone, artificial resin) (Fig. 3) [107, 110]. Natural tendon ends can be captured by custom – generally pneumatic – clamps (Figs. 4 and 6), or embedded in artificial material (Fig. 5) [56, 106]. All of these ensure that the tendon does not slip out, but both need to be monitored for the polymer to graft adhesion [56, 77–79, 106]. In those cases, the force awakening between the clamping heads ensures the success of the measurement [56, 77, 106, 107, 110] [78, 79]. Natural and artifical blocks or hydraulic presses keep the tendon in place. [107, 110].

The wedge-grip clamp and the aluminum grips with polymer liners and the strain gauge clamp are similar (Figs. 5 and 6); however, adhesion between the polymer and the tendon can be monitored [56, 106], 40,59,60]. Advantages of room temperature clamps include easy usage and no requirement for any measurement preparation. The disadvantage is that room temperature clamps can damage tendon texture, can cause the tendon to tear at the point of fixation, and the tendon can slip out.

In multiple research projects, cooled clamps are used for measuring the biomechanical properties of a tendon [42, 65, 69, 73, 108]. A great advantage of frozen clamps is that surfaces are naturally made coarse by freezing, which assists in establishing an appropriate connection between the clamp and the tendon. The solution is relatively simple: the tendon can be fastened by two metal grips fixed by screws. The first type of cooling is freezing the clamp before testing (Fig. 8). This requires a freezer that can freeze at -70ºC to -80ºC. The frozen clamp also has to be attached to the machine. The tendon takes on the clamp’s temperature over time.

The clamps shown in Figs. 9 and 10 use a dry ice container for cooling. The dry ice container allows for the tendon and the clamp to be cooled at the same time. Dry ice needs to be added during measurements, as it evaporates over time [42, 65, 73]. Both of these types of cooled clamps stop the tendon from slipping out. Cooled clamps allow for the tendon to freeze at the point of fixation, causing the tendon to tear at the weakest point [69, 108].

Heated clamps are required to be used for measurements at human body temperature (37ºC) [42, 65, 69, 73, 81, 104, 108, 114]. Leading-edge measurement designs (Fig. 13) can also imitate a human body environment (temperature, blood circulation). [104]. Heated clamps have the same disadvantages as room temperature clamps; the tendon can easily slip out, can be damaged by the clamp, or tear at the point of fixation [81, 104, 114].

### Limitation

This study focused on the investigation and categorization of existing clamps used in the determination of biomechanical properties. Due to the use of different tests and tendons, they were compared based on individual criteria. It is recommended that for subsequent tests, measurements be made only with refrigerated clamps. From the measurements made in this way, a meta-analysis of the results is obtained. This study provides an overview of clamps and does not represent the modernity of any method.

## Conclusions

The objective of this systematic literature review is to investigate and categorize existing clamps used in the determination of the biomechanical properties of tendons such as maximum load, maximum strength, modulus of elasticity, ultimate strain, and stiffness. A variety of clamps for use during the endurance test of tendons were categorized according to the temperature used during the measurement. The clamps are divided into three groups: room temperature, cooled and heated clamps. The collected information from the articles included name of clamp, author and date, type of clamps, type of endurance test (static or dynamic), type preloading (dynamic or static), type of tendon and measured and calculated parameters given in Table 1. summarized.

On the basis of systematic literature review, the mechanical properties determined for using with cooled clamps proved to be more reliable than room temperature and heated clamps. The main advantage is that there is no limit to the type and length of the tendon. The dry-ice clamp instead of liquid nitrogen is recommended for the clamping of tendons, because dry ice is cheaper to acquire than liquid nitrogen. Liquid nitrogen evaporates faster than dry ice. It is also easier to work with dry ice, permission is not needed for use, and it does not need to be stored in a container. In similar quantities, liquid nitrogen is colder than dry ice, which can harden the whole tendon, not just at the point of fixation.

Disadvantages of room temperature and heated tendons are that they can damage the tendon’s texture and have a greater chance of slipping. During the measurement, a great force is created at capture, therefore an inaccurate result can be obtained. In the case of heated clamps, it should be taken into account that living tissue, when removed from the cadaver, begins to decay. This decay can be accelerated by the warm environment, which can lead to a distortion of the results. Since there is no unlimited amount of human tissue available, the most accurate measurement setup should be used [118–121].

## Supplementary Information

**Additional file 1: **Emphasis.

## Data Availability

The data that support the findings of this study are available from authors of not open access journals but restrictions apply to the availability of these data, which were used under license for the current study, and so are not publicly available. Data are however available from Denes Farago upon reasonable request and with  permission of authors of not open access journals. All data generated or analysed during this study are included in this published review.

## Abbreviations

ACL: Anterior cruciate ligamen
